# 3′sialyllactose and 6′sialyllactose enhance performance in endurance‐type exercise through metabolic adaptation

**DOI:** 10.1002/fsn3.3559

**Published:** 2023-07-18

**Authors:** Jesus Arellano Spadaro, Yukihiro Hishida, Yukihisa Matsunaga, Monique van Es‐Remers, Henrie Korthout, Hye Kyong Kim, Eefje Poppelaars, Hiskias Keizer, Eva Iliopoulou, Bert van Duijn, Marjolein Wildwater, Lotte van Rijnberk

**Affiliations:** ^1^ Vivaltes B.V. Bunnik The Netherlands; ^2^ Kyowa Hakko Bio Co., Ltd. Tokyo Japan; ^3^ Fytagoras B.V. Leiden The Netherlands; ^4^ Institute Biology Leiden Leiden University Leiden The Netherlands

**Keywords:** *Caenorhabditis elegans*, endurance, exercise, metabolism, sialyllactose

## Abstract

Human milk oligosaccharides (HMOs) belong to a group of multifunctional glycans that are abundantly present in human breast milk. While health effects of neutral oligosaccharides have been investigated extensively, a lot remains unknown regarding health effects of acidic oligosaccharides, such as the two sialyllactoses (SLs), 3′sialyllactose (3′SL), and 6′sialyllactose (6′SL). We utilized *Caenorhabditis elegans* (*C. elegans*) to investigate the effects of SLs on exercise performance. Using swimming as an endurance‐type exercise, we found that SLs decrease exhaustion, signifying an increase in endurance that is strongest for 6′SL. Through an unbiased metabolomics approach, we identified changes in energy metabolism that correlated with endurance performance. Further investigation suggested that these metabolic changes were related to adaptations of muscle mitochondria that facilitated a shift from beta oxidation to glycogenolysis during exercise. We found that the effect of SLs on endurance performance required AMPK‐ (*aak‐1/aak‐2*) and adenosine receptor (*ador‐1*) signaling. We propose a model where SLs alter the metabolic status in the gut, causing a signal from the intestine to the nervous system toward muscle cells, where metabolic adaptation increases exercise performance. Together, our results underline the potential of SLs in exercise‐associated health and contribute to our understanding of the molecular processes involved in nutritionally‐induced health benefits.

## Graphical abstract

3′sialyllactose (3′SL) and 6′sialyllactose (6′SL) decrease exhaustion after endurance exercise in nematodes; 3′SL and 6′SL cause changes in metabolism during exercise, with a switch from beta oxidation to glycogenolysis; The increase in endurance performance requires AMPK and adenosine receptor signaling; Sialyllactoses likely signal from the intestine to the nervous system toward muscle cells, where metabolic adaptation increases exercise performance.

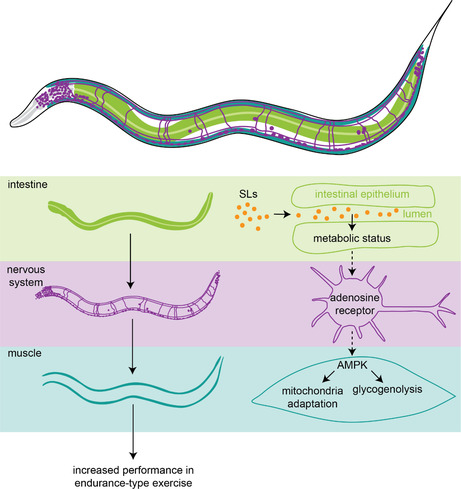

## 1 INTRODUCTION

Human milk oligosaccharides (HMOs) belong to a group of multifunctional glycans that are abundantly present in human breast milk, but largely absent from most infant formulas and present at very low concentrations in bovine milk (Zivkovic & Barile, 2011). Many infant health benefits have been ascribed to HMOs, with a clear role in shaping the infant gut microbiota and intestinal mucosal immune system (Zhang et al., 2022), and their potential for applications in adult health has been gaining attention recently (Wiciński et al., 2020). While health effects of neutral oligosaccharides have been investigated quite extensively, a lot remains still unknown regarding health effects of acidic oligosaccharides, such as sialyllactoses (SLs) (ten Bruggencate et al., 2014).

SLs consist of a lactose bound to sialic acid, either via an α‐2,3 binding (3′sialyllactose, or 3′SL), or an α‐2,6 binding (6′sialyllactose, or 6′SL), of which 6′SL is the most abundant form in human milk. Various studies in mammalian systems have identified potential effects of SLs on brain‐, gut‐, and immune health (Baek et al., 2021; Cho et al., 2021; Duan et al., 2022; Fuhrer et al., 2010; Günther et al., 2020; Kim et al., 2019; Monaco et al., 2018; Obelitz‐Ryom et al., 2019; Perdijk et al., 2019; Pisa et al., 2021; Sakai et al., 2006; Sodhi et al., 2021; Tarr et al., 2015). A recent study also found that, in mice offspring, exercise‐induced increases in milk 3′SL levels cause improvements in metabolic health and cardiac function—two major contributors of physical fitness (Harris et al., 2020). These findings indicate that SL supplementation might enhance exercise performance in general.

To investigate the potential effects of SL supplementation on physical fitness and exercise performance, we used the *Caenorhabditis elegans* (*C. elegans*) model system. The small size (up to 1 mm) and rapid growth (generation time of 3 days) of this free‐living roundworm allows high‐throughput analysis of several aspects of organism health, as well as an in‐depth investigation of conserved molecular mechanisms that underlie observed organism‐level effects.

We found that SLs improved the performance of animals in endurance‐type exercise, decreasing exhaustion after a 120‐min swim session. Using a metabolomics approach, we identified metabolic changes during exercise in animals supplemented with SLs. Further investigation indicated that supplementation of SLs caused a metabolic adaptation in which mitochondrial morphology was altered, allowing more efficient energy usage and a switch from beta oxidation to glycogenolysis. Finally, we show that AMPK and adenosine receptor signaling are involved in the effect of SLs on endurance. We propose that the effect of SLs on exercise performance is caused by a change in metabolic status in the intestine, which signals the nervous system toward muscle cells to induce metabolic adaptation. Together, these results underline the potential of SL supplementation in increasing exercise‐associated health.

## 2 MATERIALS AND METHODS

### 2.1 Nematode culture and strains

*C*. *elegans* strains were maintained at 20°C on nematode growth medium (NGM) agar plates seeded with *Escherichia coli* strain OP50 according to standard protocol unless indicated otherwise. N2 Bristol were used as wildtype. *C. elegans* strains used in this study are described in Table S1. For supplementation of animals with 3′SL, 6′SL, sialic acid (provided by Kyowa Hakko Bio Co.), lactose (Sigma CAS#: 5989‐81‐1), or lactate (Sigma CAS#: 50‐21‐5), 0.2, 1, or 2 mg/mL of compound dissolved in MQ was mixed with OP50 bacteria with an OD_600_ = 0.700–1.000. OP50 containing 3′SL or 6′SL was then added to the NGM plates and left to grow overnight before exposing nematodes. 3′ sialyllactose sodium salt and 6′ sialyllactose sodium salt were kindly provided by *Kyowa Hakko Bio Co*., *Ltd*, *Tokyo*, *Japan*. For experiments, worms were synchronized in the L1 stage by isolation of eggs from adult hermaphrodites through alkaline hypochlorite treatment and overnight hatching in M9‐Tween medium.

### 2.2 Endurance assays

Endurance assays were performed as reported previously (Laranjeiro et al., 2017), with several adaptations. In short, adult animals were placed in M9 buffer for 120 min to induce swimming exercise. Afterward, animals were placed on an NGM plate and allowed to crawl for 5 min, after which the distance or speed of their movement was determined. See Supplemental Methods (section ‘*Endurance assays*’) for a detailed protocol description.

### 2.3 Metabolomics

Metabolomics samples of three times 10,000 animals per condition per triplicate were harvested immediately after 120 min of swimming exercise. Nematodes were freeze‐dried, homogenized, and processed separately for each triplicate for GC–MS analysis on a 7890A gas chromatograph equipped with a 7693 automatic sampler coupled to a 5975C mass single‐quadrupole detector (Agilent). See Supplemental Methods (section ‘*Metabolomics*’) for a detailed protocol description.

### 2.4 Microscopy

For visualization of body wall muscle mitochondrial GFP (SJ4103), body wall muscle fat droplets (XD1875), and *tph‐1*::*GFP* expression (GR1333), synchronized L1 larvae were cultured (16°C for 96 h or 20°C for 72 h for muscle morphology, 20°C for 48 h for *tph‐1* expression), on 6‐cm agar plates in the absence or presence of 3′SL or 6′SL. For *tph‐1* expression assays, starvation was induced by placing animals on an NGM plate without bacteria for 48 h at 20°C, and animals were allowed to recover from starvation on a plate with bacteria for 4 h at 20°C. For imaging, animals were mounted on 3% agarose pads and immobilized using 1 μL of 10 mM muscimol. Images of body wall muscle mitochondrial GFP were taken using an Olympus IX71 inverted microscope with Olympus DP73 Camera, in the GFP channel (488 nm) at 40× magnification without binning, with exposure time between 200 and 400 ms, depending on signal intensity. Images of body wall muscle fat droplets were taken using a Zeiss Axioplan 2 microscope with Axiocam 305 color camera, in the GFP channel (488 nm) at 40× magnification without binning, with an exposure time of 1.8 s.

For visualization of glycogen storage, 10 μL containing +/− 30 animals were transferred to a 6‐cm NGM plate. The NGM plate was then placed over a dish of 1‐g Iodine pellets (Sigma CAS# 7553‐56‐3) for 90 s and staining was analyzed within 5 min. Images were taken using a Leica S9D dissection microscope mounted with a Leica Flexacam C1 camera in the brightfield channel. Image analysis was performed using Fiji/ImageJ. For measurements of glycogen levels, a region of interest was drawn in the midpart of the animal, just anterior of the pharynx. Staining intensity was measured by mean intensity in a grayscale image (with signal inversion: 255—intensity).

### 2.5 Oxygen consumption rate measurements

Oxygen consumption rate measurements were determined by using the seed respiration analyzer (SRA) equipment developed by Fytagoras B.V. The SRA determines the total oxygen consumption rate in a closed liquid environment. For oxygen consumption measurements, synchronized L1 larvae were grown at 16°C for 72 h in the absence or presence of 2 mg/mL 3′SL or 2 mg/mL 6′SL. Approximately 80 L4 larvae in 30‐μL M9 buffer were incubated in a tailor‐made 48‐well plate with an internal volume of 30 μL each well. After filling, the plate was closed with an airtight cover and the oxygen levels were measured immediately after covering the plate every 5 min for 1 h. See Supplemental Methods (section ‘*Oxygen consumption*’) for a detailed protocol and manifold description.

### 2.6 Pharyngeal pumping

Pharyngeal pumping was measured as previously reported (Raizen et al., 2012). In short, synchronized L1 larvae were grown to adulthood at 20°C for 72 h in the absence or presence of 0.2, 1, 2 mg/mL 3′SL, or 6′SL. Pharyngeal pumping (neuromuscular contraction in the presence of food) was assessed by counting the number of contractions within 15 s under a dissection microscope.

### 2.7 Heat stress survival

Synchronized L1 larvae were grown to adulthood at 16°C for 96 h on 6‐cm agar plates in the absence or presence of 0.2, 1, 2 mg/mL 3′SL or 6′SL. Animals were incubated in a 96‐well plate in a volume of 100‐μL S‐medium containing 2‐μM SYTOX™ Green (Catalog # S7020). Animals were then heat shocked at 34°C for 4 h in an incubator, followed by a recovery period of 20 h at 20°C. To assess survival, the number of animals showing voluntary movement (alive) vs. animals stained with SYTOX green (dead) was scored under a Leica M165FC fluorescent binocular, in the GFP channel (488 nm) at 0.73× magnification with an extra attachment of 0.5× magnification.

## 3 RESULTS

### 3.1 Sialyllactoses improve performance in endurance‐type exercise

**FIGURE 1:**
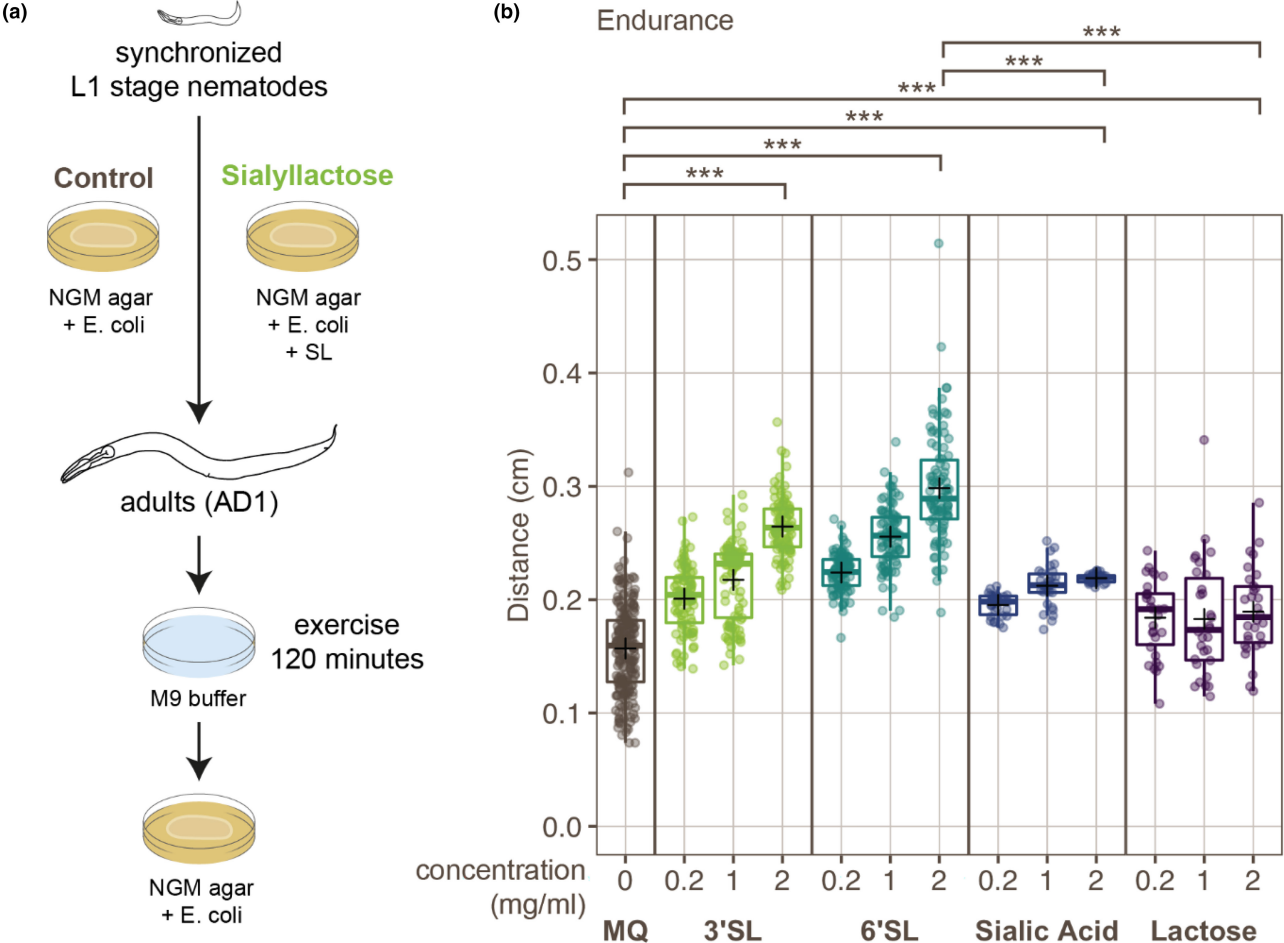
Supplementation of 3′sialyllactose or 6′sialyllactose alleviates exhaustion after swimming exercise. (a) Schematic overview of experimental procedures. Synchronized animals were grown in the absence or presence of SLs until the adult stage. Adult animals were subjected to swimming exercise by placing them in M9 buffer for 120 min, after which they were placed on an NGM agar plate seeded with *Escherichia coli* to measure movement during recovery. For further experimental procedures, see Section 2. (b) Tukey boxplots and individual points depicting the total distance crawled within 5 min of recovery time after swimming exercise, in animals grown under control conditions (MQ, *n* = 210), or in the presence of 3′SL (*n* = 90), 6′SL (*n* = 90), sialic acid (*n* = 30), or lactose (*n* = 30). Means are indicated by a black plus sign. Significance was tested by Mann–Whitney *U*‐tests with Bonferroni–Holm multiple‐comparisons correction, ***p ≤ .001.

To investigate the effects of SLs on exercise performance, we used a previously established assay to test exhaustion after endurance‐type exercise (Laranjeiro et al., 2017) (Figure 1a). In this assay, animals are placed in liquid for 120 min to induce swimming. After swimming, animals are transferred to agar plates, where the distance crawled after exercise is measured as a performance indicator, representing endurance. Animals subjected to this type of swimming exercise crawl a shorter distance than animals kept on agar plates, indicating exhaustion (Figure S1a). Interestingly, in animals grown in the presence of either 3′SL or 6′SL during their development until adulthood, we observed a dose‐dependent increase in movement after exercise, signifying an increase in endurance in which 6′SL shows a bigger increase than 3′SL (Figure 1b). To differentiate between the effect of SL and its molecular components (sialic acid and lactose), we also tested whether either one of these individual metabolites could be responsible for the observed endurance effect. We found that supplementation either with sialic acid or lactose alone caused only a slight increase in endurance, insufficient to fully explain the improvement of endurance caused by SLs (Figure 1b). In addition, the increase of endurance caused by sialic acid or lactose was not found in aging nematodes, and lactose supplementation also failed to show a significant effect in animals grown at lower temperatures (Figure S1b,c). In contrast, the endurance effect of SLs themselves was very robust and could also be observed in aging animals, animals grown at lower temperatures, and in the absence of food after exercise (Figure S1b–d). Together, these results show that SLs themselves, rather than their individual constituent metabolites, cause an improvement in endurance in exercise. Furthermore, we found that SLs did not affect animal growth or development rate and that the effect of SLs on exercise performance was not correlated with their food intake (Figure S2a,b,d,e). These results confirm that increased endurance was not a secondary effect caused by alterations in these characteristics. Interestingly, locomotion of animals supplemented with SLs was also increased under normal growth conditions (Figure S2c), indicating an adaptation caused by 3′SL and 6′SL that allowed overall higher levels of activity. We also found that 3′SL, but not 6′SL, influences survival after heat stress (Figure S3a). This indicates that these two SLs, at least in part, show distinct activities and that, while 3′SL has a smaller effect on endurance, it seems to have a bigger effect on the animals' robustness against a stress response. Nonetheless, when looking at starvation stress, both 3′SL and 6′SL showed a decreased sensitivity to starvation compared to controls (Figure S3b), which is in line with a general effect on energy metabolism.

### 3.2 Sialyllactoses induce metabolic changes during endurance exercise

**FIGURE 2:**
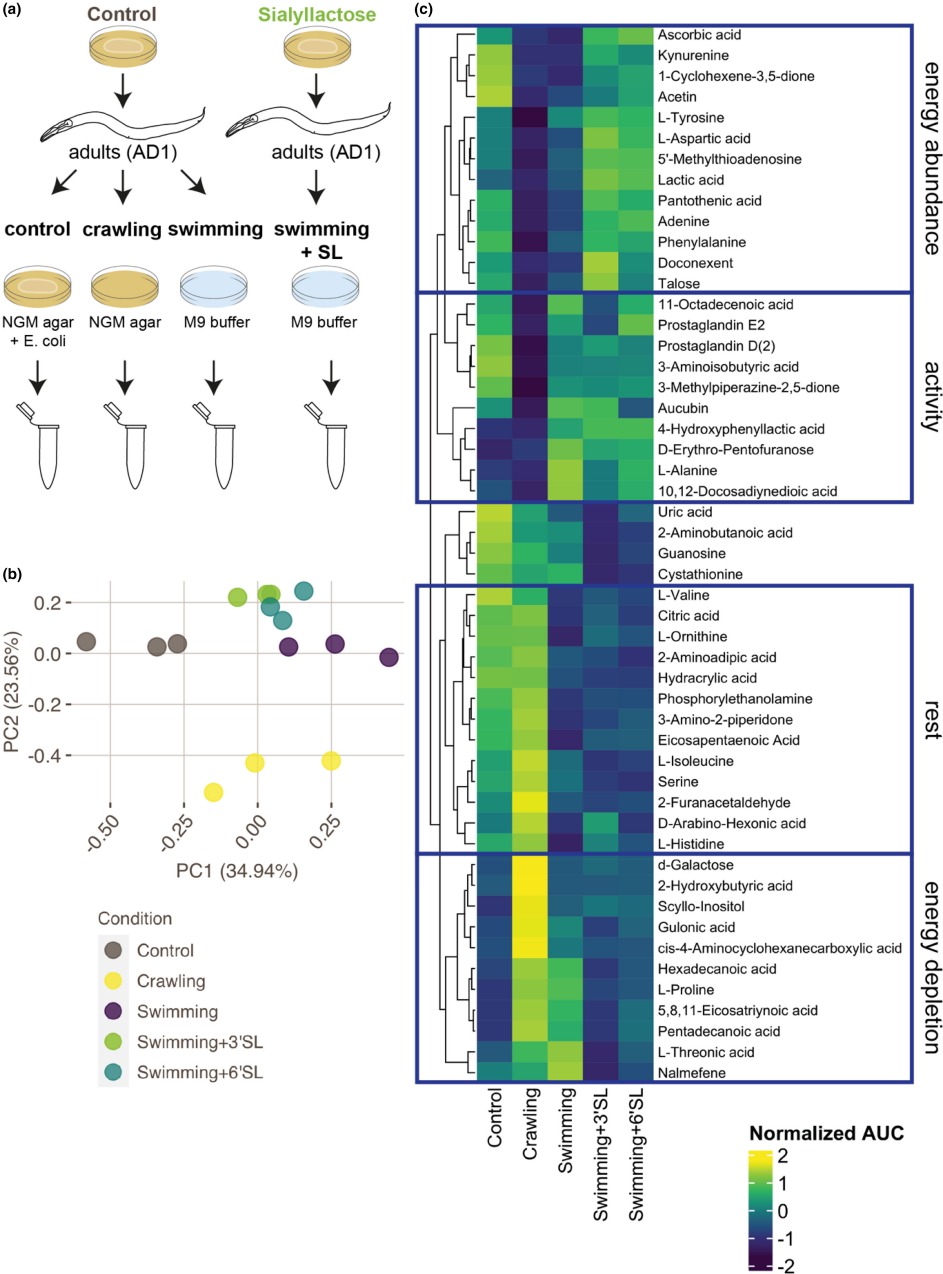
Metabolomics profiling demonstrates alleviation of energy‐related metabolic changes in animals supplemented with sialyllactoses. (a) Schematic overview of experimental procedures. Adult nematodes were grown in the absence or presence of 2 mg/mL SL and placed on control plates (control), plates without food (crawling) or subjected to swimming exercise (swimming) for 120 min. Next, animals were collected and freeze‐dried for GC–MS metabolomics analysis. (b) Principal component analysis (PCA) plot of principal component 1 (PC1) and 2 (PC2) for metabolomic profiles of animals grown in control, crawling, swimming, swimming +3′SL or swimming +6′SL conditions. Analysis includes three replicates of 10,000 animals per condition. Percentages for PC1 and PC2 indicate the proportion of the variation in the data that is explained by the principal component. (c) Heatmap depicting the normalized area under the curve (AUC) for the 56 metabolites with the biggest contribution to principal component 2 (PC2, top 50%) from metabolomics analysis of animals grown in control, crawling, swimming, swimming +3′SL or swimming +6′SL conditions. Values represent the normalized average of three replicates of 10,000 animals per condition. Metabolites are ordered by similarity in pattern, ordering shown by dendrogram. Groups of metabolites with a similar pattern across samples (manually identified) are indicated by a blue border.

To investigate whether metabolic changes might contribute to the improvement of endurance by 3′SL and 6′SL, we analyzed metabolic status after exercise in animals supplemented with SLs using an unbiased metabolomics approach (Figure 2a). Similar to aforementioned measurements of endurance, animals grown in the absence or presence of SLs were subjected to swimming exercise for 120 min. Control animals were instead kept on plates with or without an *E. coli* bacterial food source (control and crawling conditions, respectively). Next, animals were harvested by freeze‐drying samples of 10,000 animals (biological triplicates), which were subsequently used for metabolomics profiling by GC–MS. After normalization, peak identification, and filtering (see Section 2 for experimental and analysis procedures), we performed a principal component analysis (PCA, Figure 2b). In this analysis, animals supplemented with 3′SL or 6′SL were very similar and differed mostly from swimming animals along the principal component 2 (PC2) axis. To further inspect patterns of relevant metabolites, we selected the compounds represented by PC2 (56 compounds, top 50% features based on loadings, Figure 2c). Within the list of these compounds, we could distinguish four different relevant groups of metabolites with similar patterns among conditions. Two of these groups were correlated with exercise activity, where metabolite levels showed an increase (labeled ‘activity’) or a decrease (‘rest’) in samples that were subjected to exercise. Next, we identified two groups of metabolites that correlated with energy, where metabolites increase in well‐fed controls and SL‐supplemented animals (‘energy abundance’) or in starved crawling and swimming conditions (‘energy depletion’). For animals grown in the presence of SLs, we saw no clear differences in the groups of metabolites associated with rest or activity, while we did see changes in metabolites in the energy‐related groups. Among the metabolites related to energy abundance, several have previously been associated with exercise performance (such as ascorbic acid, kynurenine, phenylalanine, and tyrosine) (Higgins et al., 2020; Martin et al., 2020; Tumilty et al., 2011; Ueda et al., 2017), consistent with the results of our swimming assay. These results show that increased exercise performance is correlated to changes in metabolism during exercise in animals grown in the presence of SLs and suggest that SL supplementation causes adaptations in animals that allow them to better cope with the energetic burden of prolonged exercise on metabolism.

### 3.3 Sialyllactoses cause adaptation of muscle cell mitochondria

**FIGURE 3:**
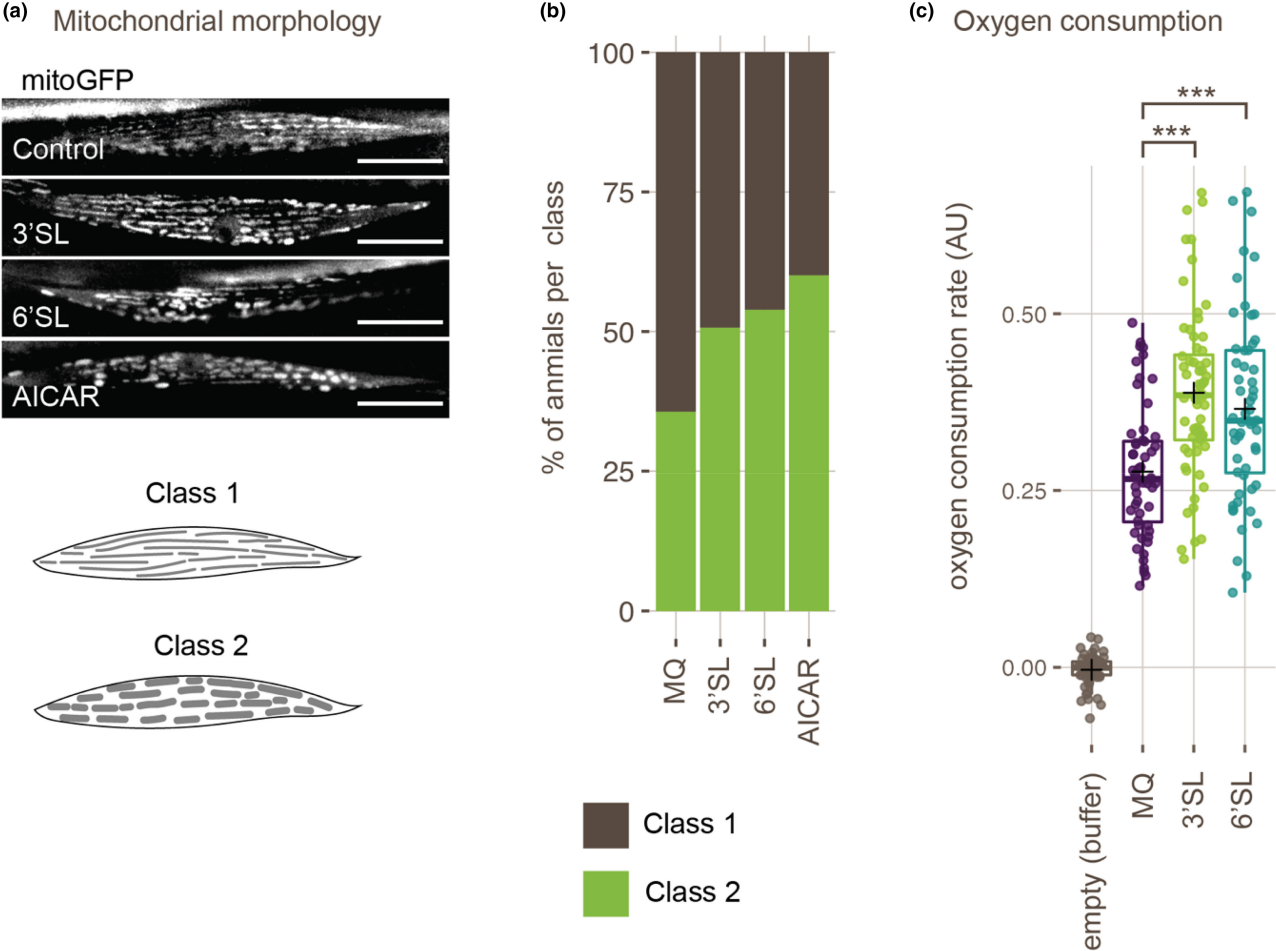
Sialyllactose supplementation causes adaptation of mitochondria. (a, b) Representative images and bar graph representing morphology of mitochondria in body wall muscle cells (% of animals per class) by analysis of mitoGFP expression in young adult animals grown under control conditions (*n* = 73), in the presence of 2 mg/mL 3′SL (*n* = 79), 2 mg/mL 6′SL (*n* = 76), or after 24‐h incubation with 1 mM AICAR (*n* = 25). Mitochondrial morphology is classified as thin and linear (class 1) or clustered structures (class 2). Scale bars are 20 μm. (c) Tukey boxplots and individual points depicting the total rate of oxygen consumption measured in each well in an air‐tight closed system over a period of 60 min. The wells contained only M9 buffer (*n* = 62), and wells contained 80 animals grown under control conditions (MQ, *n* = 56) or grown in the absence of 2 mg/mL 3′SL (*n* = 64) or 6′SL (*n* = 56). Means are indicated by a black plus sign. See Section 2 for experimental procedures. Significance was tested by Welch test with Bonferroni–Holm multiple‐comparisons correction, ***p ≤ .001.

To investigate whether metabolic adaptations could underlie the increased exercise performance induced by SLs, we decided to further investigate the body wall muscle cells, the functional equivalent of vertebrate skeletal muscles and responsible for locomotion in *C. elegans*. One type of cellular adaptation that can enhance exercise performance is a change in mitochondrial volume, density, or morphology. As the majority of energy metabolism takes place in mitochondria, it is no surprise that increases in biogenesis and changes in organization of mitochondria correlate with improvement of endurance in exercise (Lundby & Jacobs, 2016; Nielsen et al., 2017). To examine whether similar mitochondrial changes are involved in the effect of SLs on exercise performance as well, we used a mitochondrial GFP marker to visualize mitochondria in body wall muscles of control and SL‐supplemented animals (Figure 3a,b). We used a classification system, similar to previously described classifications (Hartman et al., 2018; Sarasija & Norman, 2018), to distinguish differences in mitochondrial morphology. In class 1, mitochondria are located as thin linear structures along muscle fibers, whereas in class 2, mitochondria show a more clustered morphology. As a positive control, we used AICAR, an AMPK‐activator known to boost exercise endurance and induce biogenesis of mitochondria (Fan & Evans, 2017; Hinkle et al., 2022). Although nonsignificant for any of the conditions (chi‐squared test, *p* ~ .1), we see a trend where, like AICAR, SLs showed an increase in the percentage of animals with clustered mitochondria (Figure 3b). Consistent with the effect on exercise performance, animals supplemented with 6′SL showed a bigger difference from controls than animals supplemented with 3′SL. This correlation indicates a possible link between morphological changes in mitochondria and increased endurance by supplementation with SLs.

To further investigate possible differences in mitochondrial function and efficiency, we set out to measure oxygen consumption in animals grown in the absence or presence of SLs. In a closed environment, we measured oxygen consumption of animals in liquid buffer for a duration of 1 h (Figure S4, Section 2). We found that both 3′SL and 6′SL caused an increase in basal respiration compared to untreated controls (Figure 3c). This increase in oxygen consumption could be caused by an increase in either the number or efficiency of mitochondria. Together with the change in mitochondrial morphology, these results suggest that SLs cause adaptation of mitochondria in muscle cells, which can explain the improvement in exercise performance.

### 3.4 Sialyllactoses induce a switch from beta oxidation to glycogenolysis

**FIGURE 4:**
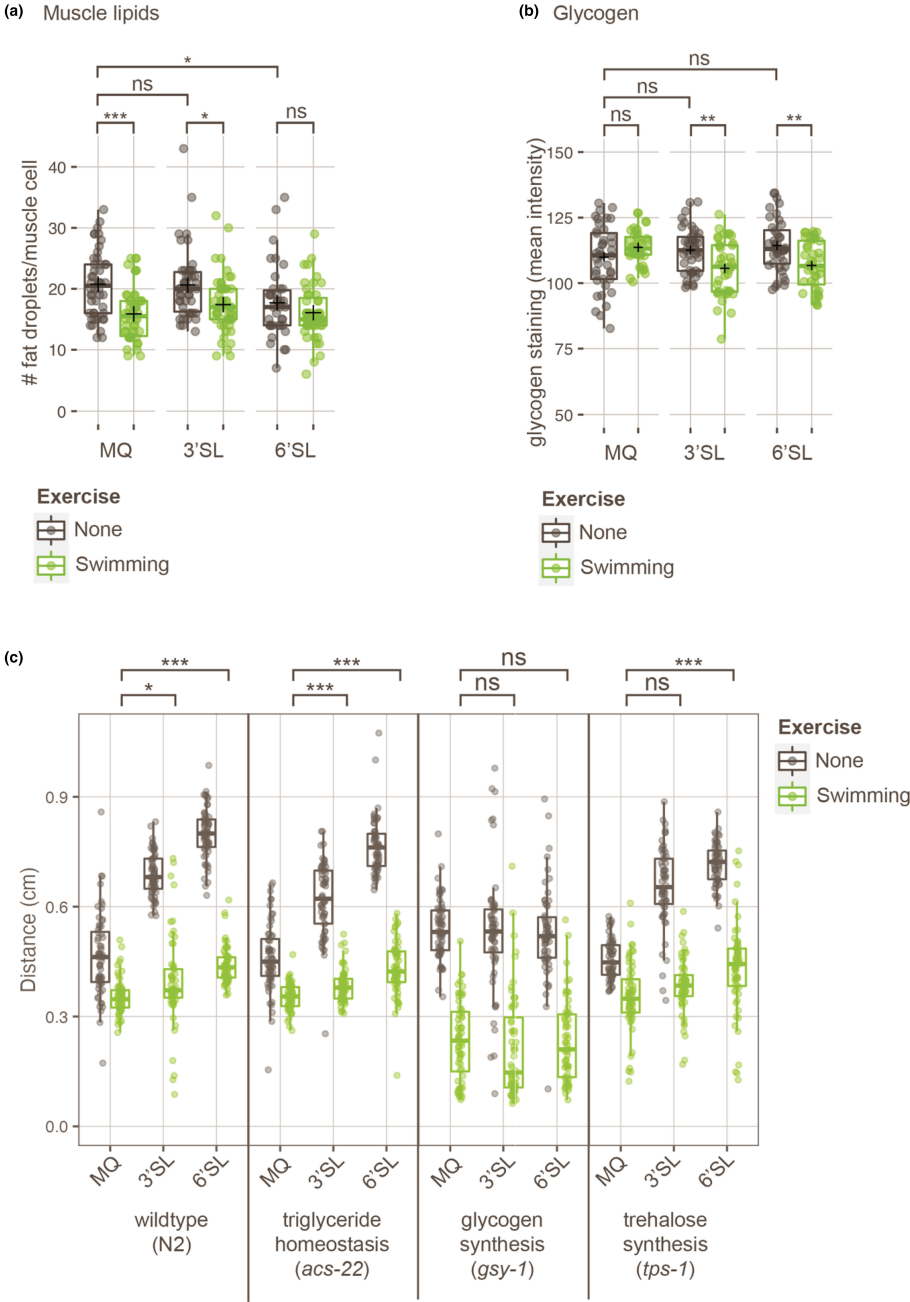
Glycogen is required for the endurance‐enhancing effect of sialyllactoses. (a) Tukey boxplots and individual points depicting the total number of lipid droplets present per muscle cell before and after swimming exercise, in animals expressing the fat droplet marker Perilipin 1‐GFP in muscle cells, grown under control conditions (MQ, *n* = 48/50, no exercise/swimming), or in the presence of 2 mg/mL 3′SL (*n* = 46/52, no exercise/swimming) or 6′SL (*n* = 38/47, no exercise/swimming). Means are indicated by a black plus sign. Significance was tested by Welch tests with Bonferroni–Holm multiple‐comparisons correction. (b) Tukey boxplots and individual points depicting the mean intensity of glycogen staining per animal before and after swimming exercise, in wild‐type animals grown under control conditions (MQ, *n* = 50/50, no exercise/swimming), or in the presence of 2 mg/mL 3′SL (*n* = 51/50, no exercise/swimming) or 6′SL (*n* = 50/50, no exercise/swimming). Means are indicated by a black plus sign. Note that the *y*‐axis does not start at zero. Significance was tested by Welch tests with Bonferroni–Holm multiple‐comparisons correction, *p ≤ .05, **p ≤ .01, ***p ≤ .001, ns *p* > .05. (c) Tukey boxplots and individual points depicting the total distance crawled within 5 min of recovery time after swimming exercise, in wild‐type (N2) or mutant background strains (*acs‐22*, *gsy‐1*, and *tps‐1*), grown under control conditions (MQ), or in the presence of 2 mg/mL 3′SL or 6′SL, and kept under standard growth conditions (None) or subjected to swimming exercise (Swimming). For each condition, 60 animals were analyzed. Significance was tested by Welch tests with FDR multiple‐comparisons correction, *p ≤ .05, ***p ≤ .001, ns *p* > .05.

Next, we set out to test for changes in the use of energy stores in muscle cells during exercise in animals grown in the presence of SLs. In wild‐type animals grown under control conditions, muscle cell fat stores are broken down during swimming exercise (Laranjeiro et al., 2017). Using a strain expressing the fat droplet marker Perilipin‐1::GFP specifically in body wall muscle cells as a marker for muscle fat storage (Laranjeiro et al., 2017; Liu et al., 2014), we quantified the number of lipid droplets per muscle cell before and after swimming exercise in animals grown in the absence or presence of SLs (Figure 4a). Interestingly, we found that animals grown in the presence of SLs—mainly 6′SL—have accumulated less fat in their muscle cells before exercise. In addition, the decrease in the number of lipid droplets after exercise is smaller in these animals, indicating a decrease in beta oxidation during exercise. With the observation of decreased use of fat stores in animals supplemented with SLs, we wondered whether these animals instead use another energy supply to fuel their swimming exercise. We, therefore, stained for glycogen and quantified the amount of glycogen in animals before and after swimming. First, we noticed that glycogen levels before exercise were similar between animals grown in the absence or presence of SLs (Figure 4b). Next, while animals grown under control conditions showed no change in the amount of glycogen after exercise, there was a significant decrease in the amount of glycogen after swimming exercise in animals grown in the presence of SLs, indicating an increase in glycogenolysis. To investigate whether energy stores of fat or glycogen, or the alternative energy source trehalose (Seo et al., 2018), are essential for the effect of SLs on exercise performance, we measured exercise endurance in mutants of fat homeostasis (*acs‐22*) (Srinivasan, 2015), glycogen synthesis (*gsy‐1*) (Seo et al., 2018), and trehalose synthesis (*tps‐1*) (Seo et al., 2018). We found that while the performance‐enhancing effect of 3′SL and 6′SL were present in mutants of fat homeostasis and trehalose synthesis, SLs did not lead to an improvement of exercise performance in a glycogen synthesis mutant (Figure 4c). These results show that supplementation with SLs causes a shift in the use of energy stores during exercise from fatty acids to glycogen and that the use of glycogen stores is essential for the endurance‐enhancing effect of 3′SL and 6′SL.

### 3.5 AMPK and adenosine receptor signaling are involved in the induction of enhanced performance in endurance‐type exercise by sialyllactoses

**FIGURE 5:**
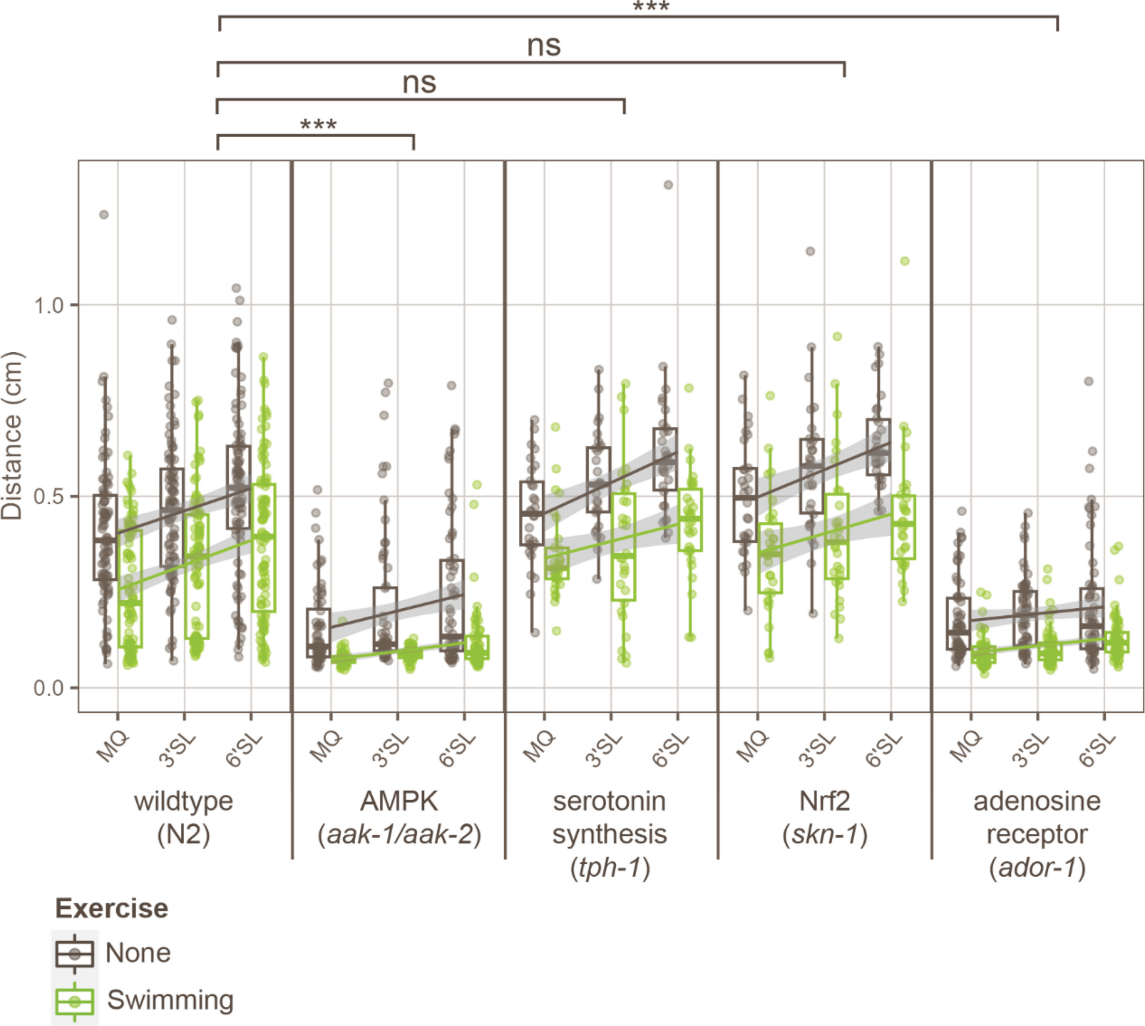
AMPK (aak‐1/aak‐2) and adenosine receptor (ador‐1) signaling are involved in the endurance increase caused by 3′sialyllactose and 6′sialyllactose. Tukey boxplots, individual points, and linear trendlines depicting the distance crawled within 5 min of recovery time after swimming exercise, in wild‐type (N2) or mutant background strains (*aak‐1/aak‐2*, *tph‐1*, *tol‐1*, *skn‐1*, and *ador‐1*), grown under control conditions (MQ), or in the presence of 2 mg/mL 3′SL or 6′SL, and kept under standard growth conditions (Exercise = None) or subjected to swimming exercise (Exercise = Swimming). For each condition, 30–90 animals were analyzed. Slopes of linear trendlines of the swimming conditions were compared between wild‐type and mutants. Significance was tested by standardized major axis tests with Bonferroni–Holm multiple‐comparisons correction, ***p ≤ .001, ns *p* > .05.

Next, we set out to identify which signaling pathways are involved in enhanced exercise performance as induced by supplementation with SLs. To do so, we measured exercise performance using our swimming assay in several mutant backgrounds of relevant pathways, such as AMPK (*aak‐1*/*aak‐2*), serotonin synthesis (*tph‐1*), Nrf2 (*skn‐1*), and adenosine receptor (*ador‐1*). AMPK is a general energy sensor, and its activation is a well‐known inducer of exercise adaptation (Spaulding & Yan, 2022). Serotonin, together with dopamine, is an important regulator of fatigue, and thereby an important factor in endurance performance (Bacqué‐cazenave et al., 2020; Sze et al., 2000). Nrf2 is a general sensor of stress and a master regulator of antioxidant defenses, which is suggested to mediate redox adaptations to exercise (Done & Traustadóttir, 2016). Finally, adenosine receptors are activated by adenosine, a signal for low levels of energy (ATP) or oxygen, and their stimulation has been linked to protection against exercise‐induced damage (Wang et al., 2010). Measuring exercise performance in animals in which these signaling pathways are blocked through mutation of an essential component allows us to test whether they are involved in the effect of SLs on exercise performance. Interestingly, we found that 3′SL and 6′SL still enhanced endurance performance in mutants for serotonin synthesis and Nrf2 signaling, but their endurance performance enhancement was significantly limited in mutants of AMPK and adenosine receptor (Figure 5), as shown by comparing slopes of trendlines using standardized major axis tests (Warton et al., 2012). Our results show that AMPK and adenosine receptor signaling are involved in the endurance‐enhancing effect of SLs, indicating that the effect on exercise performance is (partially) induced by signaling through these two pathways.

## 4 DISCUSSION

Great potential of HMOs as nutraceuticals has been proposed due to an increasing amount of evidence of their health‐beneficial effects. Here, we show that the HMOs 3′SL and 6′SL increase performance in endurance‐type exercise. Through an unbiased metabolomics approach, we found that SLs affect energy metabolism during exercise and identified changes in mitochondrial morphology and function that likely underlie this effect. We demonstrated that SLs cause a switch from fatty acid oxidation to glycogenolysis during exercise and that the endurance‐enhancing effect of SLs depends on the storage of glycogen. Finally, we found that the exercise‐beneficial effects of SLs depend on a functional adenosine receptor and AMPK signaling.

**FIGURE 6:**
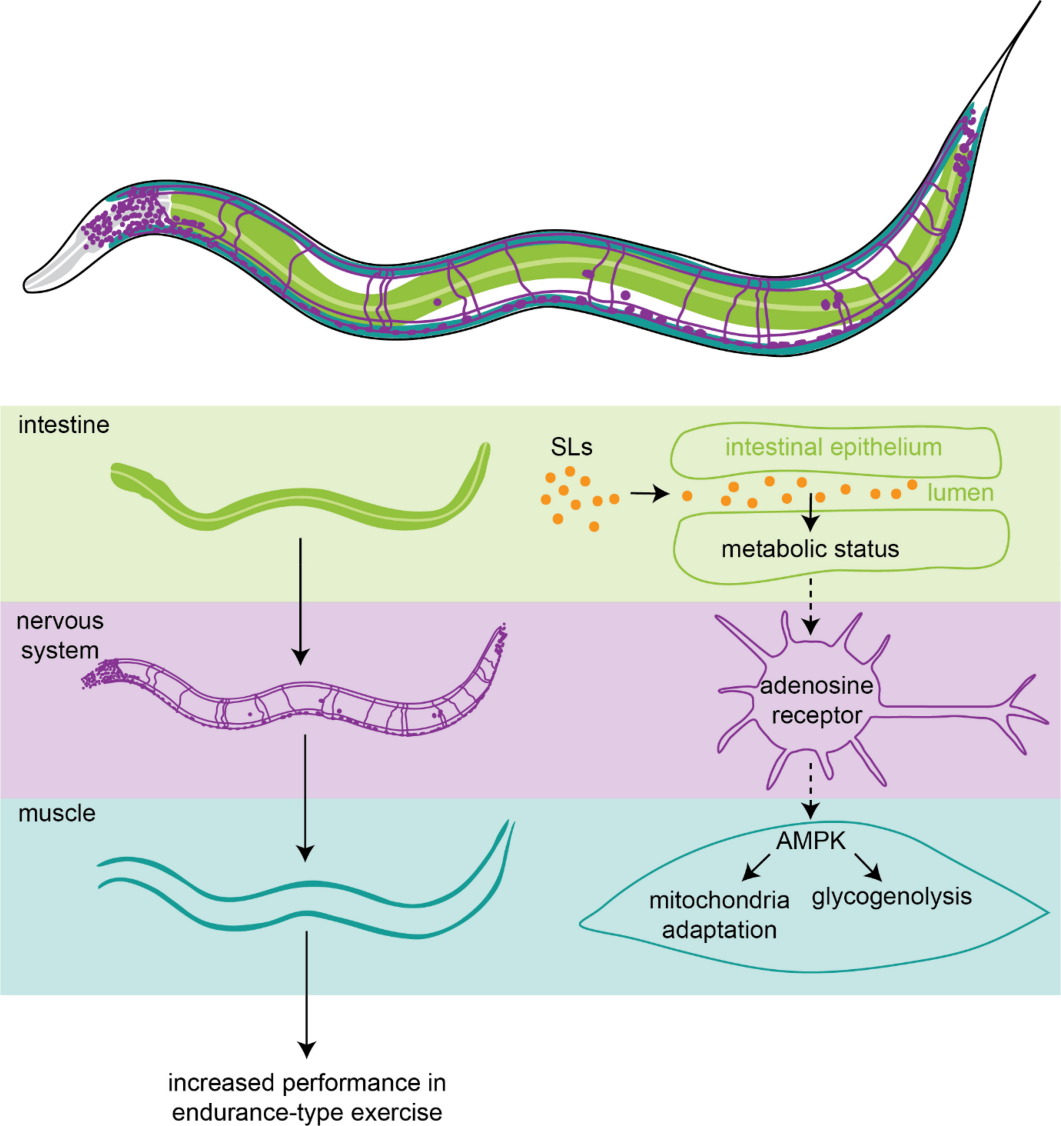
Schematic model for the induction of enhanced endurance by sialyllactoses.

The endurance effect of SLs depends on a functional adenosine receptor (*ador‐1*)—which is expressed primarily in the nervous system (Taylor et al., 2021; Von Stetina et al., 2007; Watson et al., 2008). However, we found endurance‐related adaptations in muscle cells. We propose that adaptations are induced by a signal through the gut–brain axis, which, in turn, continues toward muscle cells. A nutrient‐induced effect on (fat) metabolism of nematodes acting through ADOR‐1 has been identified previously (Machado et al., 2018), substantiating the possibility of a route from nutrient to metabolic adaptation through the gut–brain axis via the adenosine receptor. Moreover, the activity of several mammalian adenosine receptors has been linked to general muscle health and the cellular response to exercise stress (Gnad et al., 2020; Husain & Somani, 2005; Marshall, 2007), indicating that SLs might have a similar effect in humans. In addition to the adenosine receptor, we also find that AMPK is essential for the effect of SLs on exercise performance. AMPK is a general cellular energy regulator, and its stimulation is associated with an increased exercise capacity caused by cellular adaptations, largely in skeletal muscle cells (Niederberger et al., 2015). The adaptations caused by SL supplementation in our system (mitochondrial clustering, decreased fatty acid levels, and increased breakdown of glycogen during exercise) are all related to processes that are tightly regulated by AMPK (Garcia & Shaw, 2017; Hinkle et al., 2022; Janzen et al., 2018), further supporting its involvement. Together, these adaptations of muscle cells explain the improvement of performance in endurance‐type exercise caused by SLs. With this, we provide a general model of enhanced exercise performance by SLs through adenosine receptor/AMPK signaling and muscle cell adaptations (see Figure 6 for a schematic overview of the model).

Interestingly, little to no digestion of SLs occurs in the mammalian upper gastrointestinal tract (Engfer et al., 2000; Rudloff & Kunz, 2012; ten Bruggencate et al., 2014) and only very small amounts are absorbed (Galuska et al., 2020; Gnoth et al., 2001). Likewise, *E. coli*, the bacterial species that populates the nematode intestine in a laboratory setting, does not metabolize SLs (Moon et al., 2016), and nematodes lack sialic acid and its biosynthetic pathway altogether (Bacic et al., 1990; Wilson et al., 2022). We, therefore, postulate that exercise performance effects of SLs are likely caused by interactions of SLs with the intestinal epithelial cells, as suggested in previous studies on SL health effects (Duan et al., 2022; Perdijk et al., 2019; Tarr et al., 2015; Weiss & Hennet, 2012). How SLs influence intestinal health and interact with the microbiome and intestinal epithelial cells is currently not clear from this study. Additional research is needed to further substantiate if indeed the gut–brain axis is involved and if so in what manner. Previous studies using *C. elegans* have found a gut‐nervous system interaction, though the molecular mechanism is currently not identified; see review (Ortiz de Ora & Bess, 2021).

Due to technical restraints, we obtained metabolic profiles from the full organism, which is an average of many different cell types. For a more specific approach, obtaining cell‐type specific metabolic profiles from selected cell types, such as only muscle cells, would allow for a more refined analysis of the metabolites and the effect of the SLs. Additionally, Raman scattering spectrometry is a very promising tool for applying metabolomics on tissue level of *C. elegans*, e.g., to study muscle‐specific metabolic changes by SLs. However, this technology is currently only available for specific class of compounds (Fox & Schroeder, 2020). Additionally, metabolic profiles can also be influenced by the extraction and analytical procedures of metabolomics analysis (Salzer & Witting, 2021). With this study, the value of *C. elegans* in the investigation of health effects of SLs has been demonstrated, with the nematode model allowing the high‐throughput simultaneous investigation of organism‐ and tissue‐level phenotypes, intertissue communication, and molecular signaling. Overall, our results show the potential of SLs in exercise‐associated health and further our understanding of nutritionally induced health benefits.

## AUTHOR CONTRIBUTIONS

**Jesus Arellano Spadaro:** Data curation (equal); formal analysis (supporting); investigation (equal); methodology (equal); writing – review and editing (supporting). **Yukihiro Hishida:** Conceptualization (equal); funding acquisition (equal); resources (equal); writing – review and editing (supporting). **Yukihisa Matsunaga:** Conceptualization (equal); funding acquisition (equal); resources (equal); writing – review and editing (supporting). **Monique van Es‐Remers:** Data curation (equal); formal analysis (supporting); investigation (equal); methodology (equal); writing – review and editing (supporting). **Henrie Korthout:** Investigation (supporting); methodology (equal); supervision (equal); visualization (equal); writing – review and editing (equal). **Hye Kyong Kim:** Data curation (equal); formal analysis (equal); investigation (equal); methodology (equal); writing – review and editing (supporting). **Eefje Poppelaars:** Formal analysis (supporting); methodology (equal); project administration (equal); visualization (equal); writing – review and editing (equal). **Hiskias Keizer:** Investigation (supporting); methodology (supporting); writing – review and editing (supporting). **Eva Iliopoulou:** Data curation (equal); formal analysis (supporting); investigation (equal); writing – review and editing (supporting). **Bert van Duijn:** Methodology (supporting); resources (equal); supervision (supporting); writing – review and editing (supporting). **Marjolein Wildwater:** Conceptualization (equal); funding acquisition (equal); methodology (equal); project administration (equal); resources (equal); supervision (lead); writing – original draft (supporting); writing – review and editing (equal). **Lotte van Rijnberk:** Data curation (equal); formal analysis (lead); methodology (equal); project administration (equal); visualization (equal); writing – original draft (lead); writing – review and editing (equal).

## FUNDING INFORMATION

Vivaltes B.V. is an independent R&D company with seven employees. The business model is to use *C. elegans* (as well as data analytics) as a test system to study health beneficial and toxic effects of compounds including complex extracts. Vivaltes acts as an independent research organization for both industries and Universities (www.vivaltes.com). No revenues, sales activities, or other profit is made of 3′Sialyllactose and 6′Sialyllactose or its applications. No commercial benefits are gained from this publication. The research is part of the PhD work of JA. He is a shared PhD student at Vivaltes and Leiden University. All research agreements between Vivaltes and her clients furthermore are based on contracts for a fixed period of time and never depend on data results. Fytagoras B.V.: Research company with 11 employees. Business model: Exploring metabolomics technology to study health‐promoting and entourage effects in complex extracts and compound identification; Screening for industries and Universities. www.fytagoras.com. No revenues, sales activities, or other profit making from Sialyllactoses. No commercial benefits from this publication. Kyowa Hakka Bio: The role of Kyowa was to provide 3′Sialyllactose and 6′Sialyllactose as well as to participate in scientific discussions during the research period.

## CONFLICT OF INTEREST STATEMENT

Competing interests: Authors from Vivaltes and Fytagoras and Leiden University declare that they have no competing financial interests or personal relationships that could have appeared to influence the work reported in this paper. Both Vivaltes and Fytagoras are independent research organizations and as such not influenced by Kyowa. Authors from Kyowa declare that as they are owners of the Sialyllactose as such they may be considered as having potential competing interests.

## Supporting information


Data S1.

## Data Availability

Data available on request from the authors.
