# Supplementary material for: 3′sialyllactose and 6′sialyllactose enhance performance in endurance‐type exercise through metabolic adaptation
Source: Food Sci Nutr. 2023 Jul 18;11(10):6199–212. doi: 10.1002/fsn3.3559 (PMC10563706; doi:10.1002/fsn3.3559)
Supplement: Supplementary file 1 — Data S1. [file FSN3-11-6199-s001.docx]

**
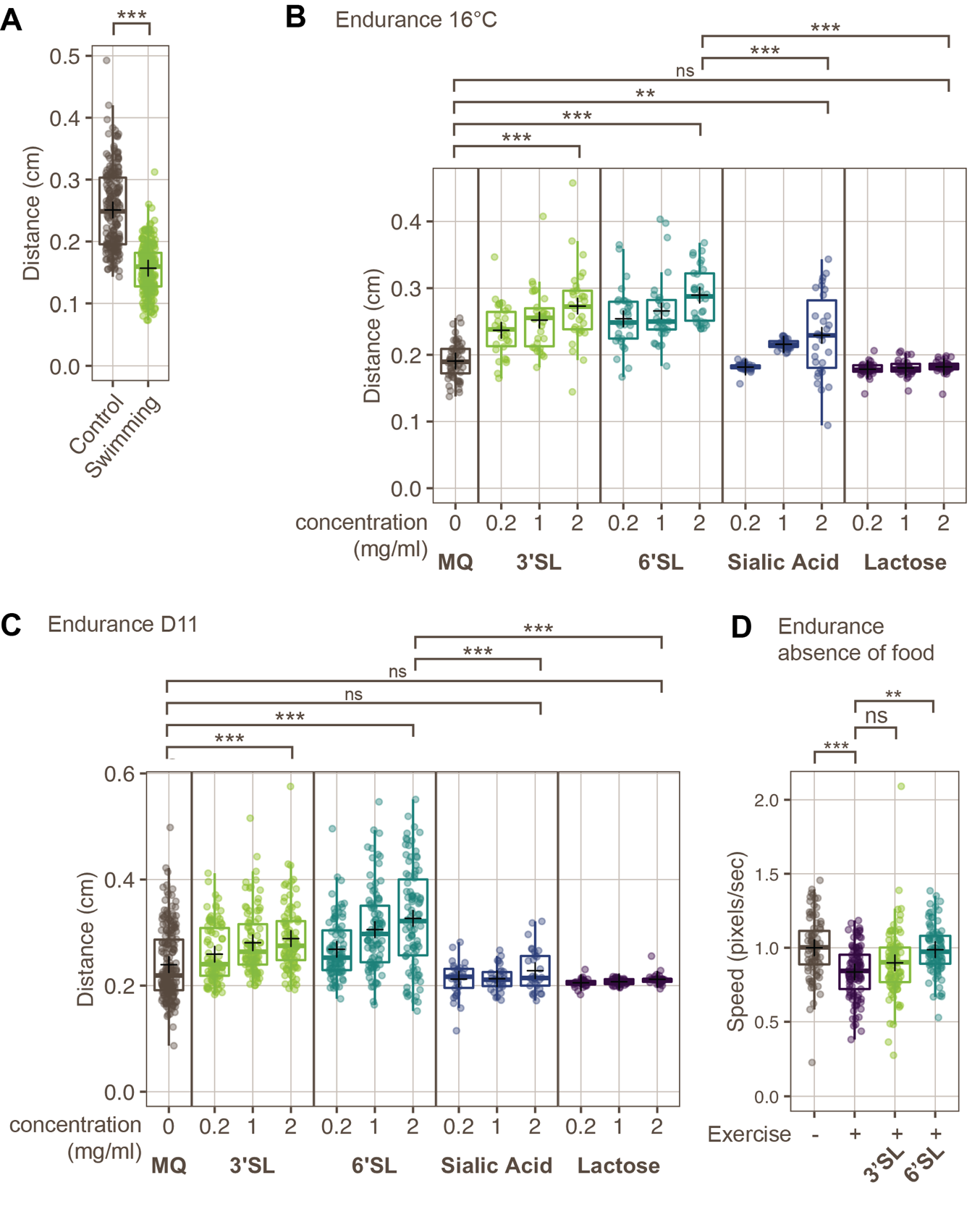
**

**Supplemental Figure 1.** *3’Sialyllactose and 6’Sialyllactose increase endurance after swimming exercise in aging animals and lower temperatures.*

**(A)** Tukey boxplots and individual points of the total distance crawled within 5 minutes for animals grown on NGM agar plates without food (Control, n = 210) or animals subjected to swimming exercise for 120 minutes (Swimming, n = 210). Means are indicated by a black plus sign. Significance was tested by Mann-Whitney U tests, *** P ≤ 0.001. **(B-C)** Tukey boxplots and individual points of the total distance crawled within 5 minutes for animals grown until young adults at 16°C, or until late adults (11 days/D11) at 20°C, either under control conditions (MQ, n = 60 and 210, respectively), or in the presence of 3’SL (n = 30 and 90), 6’SL (n = 30 and 90), sialic acid (n = 30 and 30) or lactose (n = 30 and 30). Means are indicated by a black plus sign. Significance was tested by Mann-Whitney U tests with Bonferroni-Holm multiple-comparisons correction, ** P ≤ 0.01, *** P ≤ 0.001, ns P > 0.05. **(D)** Tukey boxplots and individual points depicting the mean crawling speed (normalized to non-swimming controls) in animals grown under control conditions (MQ - non-swimming, n = 87), subjected to swimming (MQ - swimming, n = 97), grown in the presence of 2 mg/ml 3’SL and subjected to swimming (3’SL - swimming, n = 87), or 2 mg/ml 6’SL and subjected to swimming (6’SL - swimming, n = 88). In contrast to the schematic overview in panel A and the results shown in panel B, animals were placed on NGM plates without a bacterial food source after swimming exercise. Significance was tested by students *t*-tests with Bonferroni-Holm multiple-comparisons correction, ** P ≤ 0.01, *** P ≤ 0.001, ns P > 0.05.

**
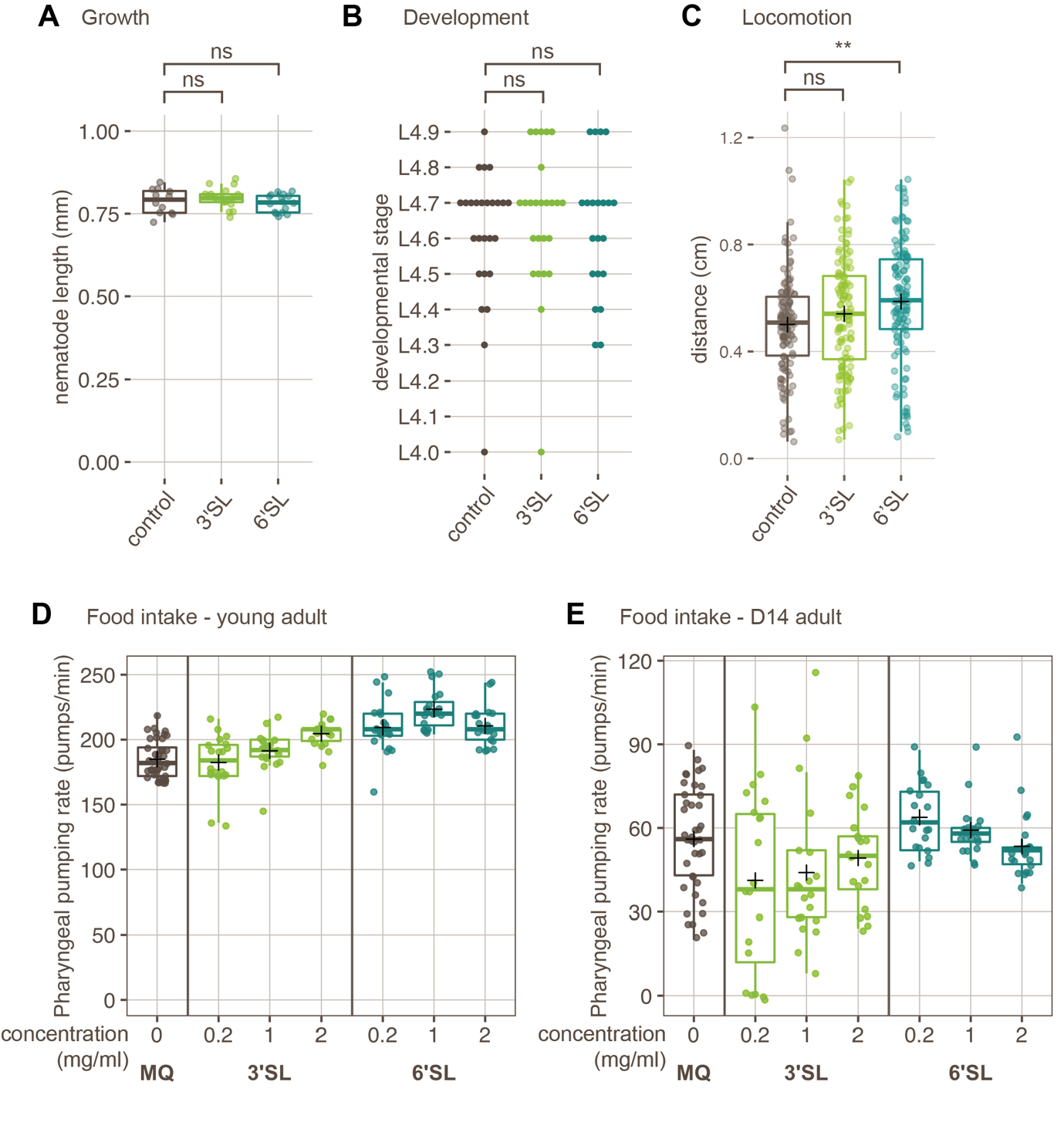
**

**Supplemental figure 2.** *The endurance effect of supplementation with 3’Sialyllactose or 6’Sialyllactose does not correlate with growth speed, development, or food intake.*

**(A)** Tukey boxplots and individual points of total nematode length after 72 hours of development at 16°C, in animals grown under control conditions (n = 12) or in the presence of 2 mg/ml 3’SL (n = 17) or 6’SL (n = 16). Significance was tested by students *t*-test with Bonferroni-Holm multiple comparisons correction, ns P > 0.05. **(B)** Dot plot of L4 developmental (sub)stage after 72 hours of development at 16°C, in animals grown under control conditions (n = 26) or in the presence of 2 mg/ml 3’SL (n = 25) or 6’SL (n = 21). Significance was tested by students’ *t*-test with Bonferroni-Holm multiple comparisons correction, ns P > 0.05. **(C)** Tukey boxplots and individual points of nematode locomotion (total distance crawled within 5 minutes) for animals grown under control conditions (n = 120), or in the presence of 2 mg/ml 3’SL (n = 120) or 6’SL (n = 120). Significance was tested by students *t*-tests with Bonferroni-Holm multiple comparisons correction, ** P ≤ 0.01, ns P > 0.05. Means are indicated by a black plus sign. **(D-E)** Tukey box plots and individual points depicting average pharyngeal pumping rate for animals grown under control conditions (MQ) or in the presence of 0.2, 1 or 2 mg/ml 3’SL or 6’SL, at the young adult stage (D) or in aging (14 day) animals (E). For each condition, 20-40 animals were analyzed. Means are indicated by a black plus sign.

**
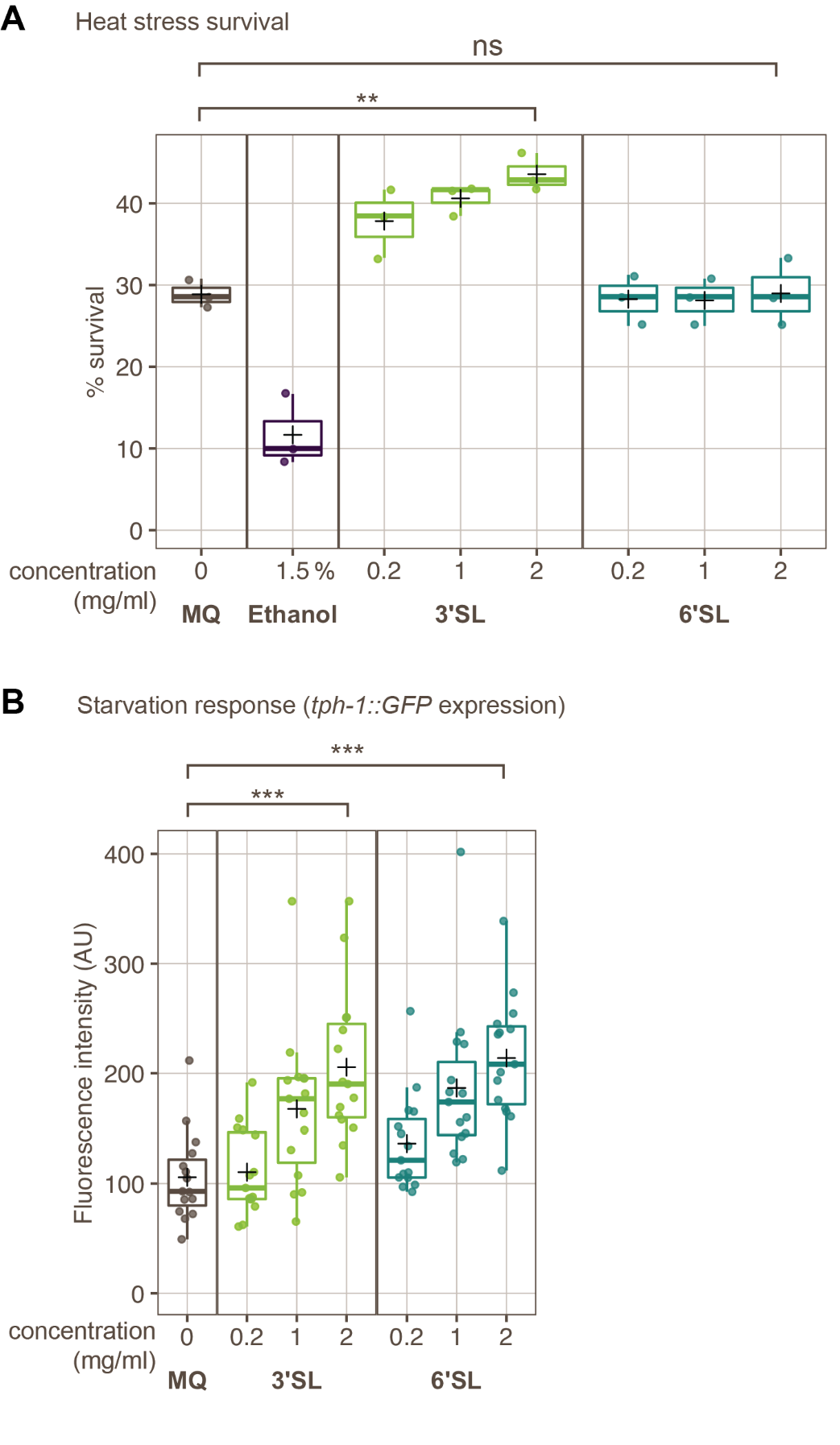
**

**Supplemental figure 3.** *Survival after heat stress is improved after supplementation with 3’Sialyllactose, but not 6’Sialyllactose.*

**(A)** Tukey boxplots and individual points depicting the percentage of surviving animals after incubation at 34°C for 4 hours. For each condition, 3 replicate plates containing 10-16 animals were analyzed. Ethanol was used as a control for decreased survival. Means are indicated by a black plus sign. Significance was tested by students *t*-tests with Bonferroni-Holm multiple comparisons correction, ** P ≤ 0.01, ns P > 0.05. **(B)** Tukey boxplot and individual points depicting average *tph-1::GFP* expression after 48 hours of starvation and 4 hours recovery in young adult animals grown under control conditions or in the presence of 0.2, 1 or 2 mg/ml 3’SL or 6’SL (n = 15 per condition). Means are indicated by a black plus sign. Significance is tested by students’ *t*-tests, *** P ≤ 0.001.


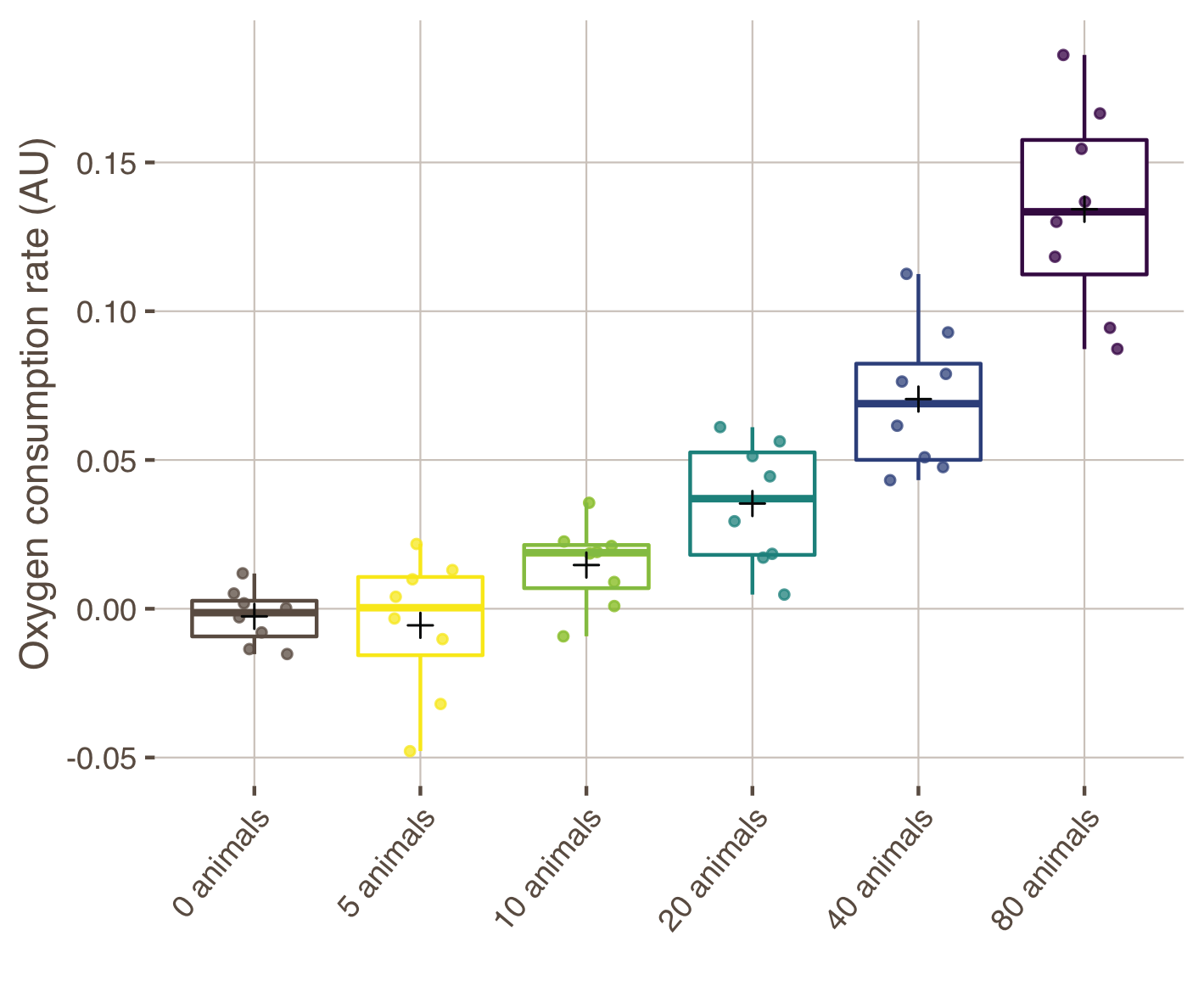


**Supplemental figure 4.** *Oxygen consumption measurements of nematodes in a closed liquid environment.*

Tukey boxplots with individual points depicting the average oxygen consumption rate of a dilution series of animals in M9 buffer, measured in a closed system for a duration of 1 hour. Means are indicated by a black plus sign.

**Supplemental figure 5**. *Design of a 48-well plate for studying the Oxygen Consumption Rate of C. elegans by using an adapted SRA (Seed Germination Analyzer).*

The manifold consists of an upper and lower part, specifically designed for measuring small volumes in an airtight environment. **(A)** The lower part of the manifold with 48 wells with an internal volume of 30 µL in each well. The inert oxygen sensitive coating (b) is in the bottom of each well, and surrounded with an NBR rubber ring (c) to ensure an airtight closure of each well after covering the lower plate with the upper part by four screws (a). **(B)** Cross section of the manifold with the upper plate (e) connected to the lower plate (f) establishing an airtight closing of the well with the NBR ring (g). The Oxygen sensitive coating is located at the bottom of the well (h) and fluorescence is measured by a detector (d).

**Supplemental Table 1. *C. elegans* strains**

| **Strain name** | **Genotype** | **Source/reference** |
| --- | --- | --- |
| SJ4103 | *zcIs14 [myo-3::GFP(mit)]* | *Caenorhabditis* Genetics Center |
| XD1875 | *xdIs26[P _unc-54_PLIN1::GFP rol-6(su1006)]* | Xun Huang lab |
| NB245 | *aak-1(tm1944) III; aak-2(gt33) X.* | *Caenorhabditis* Genetics Center |
| MT15434 | *tph-1(mg280) II.* | *Caenorhabditis* Genetics Center |
| IG10 | *tol-1(nr2033) I.* | *Caenorhabditis* Genetics Center |
| GR2245 | *skn-1(mg570) IV.* | *Caenorhabditis* Genetics Center |
| EG6890 | *ador-1(ox489) II.* | Jorgensen lab |
| QQ255 | *gsy-1(gk397885) II.* | *Caenorhabditis* Genetics Center |
| VC225 | *tps-1(ok373) X.* | *Caenorhabditis* Genetics Center |
| CB7468 | *acs-22(gk373989) X.* | *Caenorhabditis* Genetics Center |

**Supplemental Methods**

*Endurance assays*

Nematode populations were synchronized by hypochlorite treatment of gravid adult hermaphrodites to isolate individual eggs. Eggs were incubated in M9 buffer overnight to allow hatching of L1 larvae in the absence of food. Synchronized L1 larvae were cultured on NGM agar plates seeded with OP50 bacteria 20°C for 72 hours (young adult) or 11 days (aged animals), where the bacterial food source was supplemented with either MQ (control), 3’Sialyllactose, or 6’Sialyllactose (0.2, 1 or 2 mg/ml). To induce swimming exercise, animals were washed off the NGM agar plates and placed into 2 mL of M9-Tween buffer for 120 minutes. After 120 minutes of exercise, droplets of 10 µl of buffer, containing approximately 15 animals per droplet, were placed on NGM agar plates with or without OP50 bacteria and left to crawl for 5 minutes. For measurements of distance crawled, a video of the tracks of the animals in the bacterial lawn was made using a Leica S9D dissection microscope mounted with a Leica Flexacam C1 camera. The distance traveled by each animal was quantified by their tracks using Fiji/ImageJ software. For measurements of speed, a video of animals crawling on a plate without bacteria was made using a Leica S9D dissection microscope mounted with a Leica Flexacam C1 camera. The speed of each animal during a period of 20-30 seconds was quantified using Fiji/ImageJ software. To quantify the speed of the animals, videos were imported as image stacks and converted to 8-bit grayscale using the plugin “FFmpeg video”. Subsequently, the minimum intensity Z projection is used to visualize the tracks of each nematode present in the video. The tracks were selected using the “segmented line” selection tool and their length in pixels was measured with the plugin “Measure and label”. To calculate the pixels/second, the length of each track was divided by the exact time of the video.

*Metabolomics*

Nematode populations were synchronized by hypochlorite treatment of gravid adult hermaphrodites to isolate individual eggs. Eggs were incubated in M9 buffer overnight to allow hatching of L1 larvae in the absence of food. Synchronized L1 larvae were cultured at 16°C for 96 hours on 9 cm agar plates in the absence or presence of 2 mg/ml 3’SL or 6’SL in the OP50 bacterial food source. To induce swimming exercise, animals were washed off the NGM agar plates and placed into 2 mL of M9-Tween buffer for 120 minutes. For the crawling negative control, nematodes were transferred to an NGM plate without OP50 food source. Approximately 10,000 nematodes were freeze-dried and the dry weight was measured for each individual sample. Triplicate biological replicates were performed. Animals were then homogenized using a 5 mm glass bead TissueLyser II (Qiagen) for 5 min at frequency of 30 Hz. 1 ml of 80% MeOH was added and sonicated for 10 min and then centrifuged for 10 min to obtain clear extracts. The extract was dried using a Speed-Vac concentrator. Dried extracts were re-dissolved with 100 μL of pyridine containing internal standard (methyl palmitate 0.2 mg/ml). 100 μL of BSTFA + TMCS (99:1) was added, and samples were incubated at 80°C for 60 min in an incubator. The solutions were then centrifuged at 17,000 rcf for 10 min, and the supernatants were transferred to micro-inserts for GCMS analysis on a 7890A gas chromatograph equipped with a 7693 automatic sampler coupled to a 5975C mass single-quadrupole detector (Agilent, Folsom, CA, U.S.A.). Separation was performed on a DB5 GC column (30 m × 0.25 mm, 0.25 μm thickness, JW Science, Folsom, CA, U.S.A.) with helium (99.9% purity) as the carrier gas at a flow rate of 1 mL/min. The initial oven temperature was 60°C for 1 min, and then ramped at 10°C/min to 280°C, held for 1 min, and ramped again to 310°C at 8°C/min for 5 min. The injector was set at 275°C and 1 μL of each sample was injected in splitless mode. The interface temperature was 280°C, and the ion source and quadrupole temperature of the mass detector was 230°C and 150°C, respectively. Ionization energy in EI mode was 70 eV, and peaks were identified by comparison of the ion spectra with those in the NIST library (version 2008). Analysis of metabolomics profiles was performed using MZMine software and R (version 4.1.2)^[64]^. In short, peaks were identified using MZMine software and area under the curve was normalized to an internal standard and sample weight. For duplicate peaks that correspond with the same metabolite, only one peak with the highest area under the curve was included for downstream analysis, resulting in a profile of 106 unique metabolites. These metabolites were used for principal component analysis. For selection of interesting metabolites to be visualized in a heatmap, the top 50% metabolites were selected based on the feature loadings in principal component 2.

*Oxygen consumption rate measurements*

The Oxygen Consumption Rate (OCR) of *C. elegans* was measured in Q2 Respiration Analyzer (Fytagoras B.V., Leiden, the Netherlands)^[65]^. To adapt the analyzer for C. *elegans*, a tailor-made 48 well 127.8 x 85.5 mm PMMA device was developed and produced (Merrem, Dinxperlo, the Netherlands). The device consists of two PMMA plates with a thickness of 20 mm each. The upper plate contains 48 wells filled with fluoridated silicone polymer as the oxygen sensitive coating. The lower plate contains 48 wells of 30 µl each for incubation of the nematodes in M9 buffer as well as the fluoridated silicone polymer coating in the bottom of each well. Each individual well was surrounded by a 7.5 x 1.0 mm NBR O-ring (Techniparts, Wezep, the Netherlands) in order to establish an airtight connection between the nematode containing wells in the lower plate and the oxygen sensitive coating in the upper plate (see Supplemental Figure 5). Both plates were mounted to each other with four screws that were placed in each corner of the plate. The presence of the coating in the upper plate wells allows the measurement of the oxygen concentration in the headspace of the wells, whereas the presence of the coating in the lower plate wells allows the measurement of the oxygen concentration at the location of the nematodes in the bottom of the wells. The OCR was followed during 30 minutes at a controlled temperature of 21.5 ⁰C.

To measure oxygen consumption in nematodes, synchronized L1 larvae were grown until L4 stage at 16°C for 72 hours in 9-cm agar plates in the absence or presence of 2 mg/ml 3’SL or 2mg/ml 6’SL. Eighty L4 larvae in 30 μl M9 buffer were incubated in the PMMA 48-well plate sealed and oxygen measurements were performed every 5 minutes for 1 hour.
